# A Shift in Myeloid Cell Phenotype via Down Regulation of Siglec-1 in Island Macrophages of Bone Marrow Is Associated With Decreased Late Erythroblasts Seen in Anemia of Critical Illness

**DOI:** 10.3389/fmed.2019.00260

**Published:** 2019-11-20

**Authors:** Shirin Hasan, Maria Camargo Johnson, Ameet R. Kini, Anthony J. Baldea, Kuzhali Muthumalaiappan

**Affiliations:** ^1^Health Sciences Division, Department of Surgery, Loyola University Chicago, Maywood, IL, United States; ^2^Health Sciences Division, Burn and Shock Trauma Research Institute, Loyola University Chicago, Maywood, IL, United States; ^3^Health Sciences Division, Department of Pathology, Loyola University Chicago, Maywood, IL, United States

**Keywords:** Siglec 1, erythropoiesis, Ly6G, bonemarrow, spleen, erythroblast-island macrophages

## Abstract

Burn injury has been shown to significantly dampen erythropoiesis in both burn patients and in murine models. Our previous findings elucidated the erythropoietin independent defects in red cell development stages involving erythroid progenitor production and late stage erythroblast enucleation processes. We hypothesized that macrophages (MØ) in erythroblast islands (EBI) could be yet another roadblock impeding erythropoiesis following burn injury. Here we highlight that the methodology to study EBI can be achieved with single cell suspensions using a simple technique such as flow cytometry, as obtaining the central erythroblast island macrophages (EBIMØs) of interest is a delicate process. We elucidated the requisite of EBIMØ from the erythroblast as well as the MØ perspective. In addition to the primary erythropoiesis organ, the bone marrow (BM), spleens were also examined for extra-medullary erythropoiesis. Femurs and spleens were harvested from adult mice (B_6_D_2_F_1_) subjected to 15% total body surface area (TBSA) scald burn (B) or sham burn (S). Total bone marrow cells (TBM) and splenocytes were probed for total erythrons, early and late erythroblasts and EBIMØ by flow cytometry. There was only a marginal increase in the number of EBIMØ after burn, but, between the signatures of EBIMØ, Siglec-1 expression (MFI) was reduced by 40% in B with and a parallel 44% decrease in TBM erythrons in the BM. There were more (2.5-fold) EEBs and less LEBs (2.4-fold) per million TBM cells in B; with a corresponding decrease in Siglec-1 and Ly6G expressions in EBIMØ associated with EEB. Conversely, extra-medullary erythropoiesis was robust in spleens from B. Not only were the numbers of EBIMØs increased in B (*p* < 0.002), both EEBs and LEBs associated with EBIMØ were higher by 30 and 75%, respectively. Importantly, an increase in Siglec-1 and Vcam1 expressing F480^+^ splenic macrophages was observed after burn injury. Therefore, stagnant EEBs in the BM after burn injury could be due to low Siglec1 expressing EBIMØ, which perhaps impede their maturation into LEBs and reticulocytes. Repercussion of myeloid cell phenotype specific to BM after burn injury could plausibly account for a defective late stage RBC maturation resulting in anemia of critical illness.

**Summary Sentence:** Characterization of erythroblast island macrophages (EBIMØ) in the bone marrow and spleen at different stages of erythropoiesis after burn injury.

## Introduction

Anemia, immune-suppression, and monocytosis are immune consequences of a traumatic burn injury (1, 2). Furthermore, burn injury bifurcates the common myeloid progenitors toward granulocyte monocyte commitment by increasing MafB (myeloid transcription factor) expression and thereby reducing the production of megakaryocyte erythrocyte progenitors (MEP) (3–5). Monocytes that mature into tissue macrophages (MØ) also serve a unique function of nurturing the erythroblasts during erythropoiesis (6, 7). Besides the hematopoietic shortcoming of bone marrow MEP commitment, it is not known whether myeloid cells stemming from burn-induced microenvironment also impede red blood cell development. As burn injury affects both the erythroid and myeloid arms of hematopoiesis resulting in Epo resistant anemia (1, 8, 9), we utilized this burn model to study the bearing of MØ phenotype in burn-mediated anemia of critical illness. The concept of erythroblast island macrophages (EBIMØ) has been known for more than 60 years (10) but not completely understood (11, 12). Recent *in vitro* evidence using time lapse video microscopy shows the interaction of macrophages with erythroblasts at various stages of maturation (13). Therefore, characterizing the phenotype of EBIMØ in burn pathology is essential to understand the efficacy of erythroblast islands contributing to the maturation of erythroblasts and to eventually underscore the intercellular signals.

For the characterization of EBIMØ, emerging studies point to multiple markers such as CD163, Ly6G, ERHR3, Vcam1, and Siglec1 associated with F4/80^+^ macrophages (6, 14–17). Due to the structural arrangement of an EBI in which the central macrophage is surrounded by immature erythroblasts, techniques like immunohistochemistry and immunofluorescence do not provide both qualitative and quantitative information required for studying the changes occurring in these macrophages under different pathological conditions, while use of flow cytometry has provided significant information in the characterization of EBIMØs (17, 18). Therefore, in this study we predominantly screened EBIMØs using eight-color flow cytometry to comprehend the interference of macrophage abnormality during erythroblast maturation after burn injury. Moreover, under hypoxic conditions of burn injury, the spleen serves as an extra medullary erythropoietic organ in adult mice. Therefore, to compare and contrast erythropoiesis between the two organs in response to burn injury we also examined the phenotype of EBIMØ, the distribution of erythroblasts and RBCs in spleen. Of the two organs, EBIMØs in the spleen facilitate erythroblast maturation contributing to extra medullary erythropoiesis where as, bone marrow EBIMØ phenotype is certainly compromised resulting in the inhibition of erythroblast maturation.

## Methods

### Mouse Model of Burn Injury

#### Mice

Six to eight weeks old B6D2F1 male mice weighing ~25 g were purchased from Jackson Laboratories (Barr Harbor, ME). Mice were housed in our Comparative Medicine Facility with 12 h light/dark cycle with controlled temperature (20–22°C). The mice were allowed to acclimate to our facility for 7 days prior to use. The Institutional Animal Care and Use Committee at Loyola University Medical Center approved all experimental protocols.

### Burn Injury

B6D2F1 male mice were randomly divided into sham and burn groups. Mice were anesthetized using ketamine and xylazine (100, 2.5 mg/kg, respectively; intraperitoneal) and their dorsal hair was removed by shaving. Mice were subjected to a 15% TBSA full thickness scald burn along the dorsum by immersion in a 100°C water bath for 8 s (19) and were resuscitated immediately with intra-peritoneal injection of normal saline (2 mL). A sham group of mice were administered anesthesia, shaved and resuscitated but were not subjected to burn injury. Animals were sacrificed either on post burn days 3, 7, 14, or 21 as indicated in the results section. All experiments were repeated four to five times.

### Isolation of Total Bone Marrow Cells and Splenocytes

Total bone marrow cells from the bilateral femurs of each mouse were eluted into McCoy's medium (Invitrogen, Carlsbad, CA, USA) using a 25-gauge needle. Spleens were mashed with the back of a 3 ml syringe plunger and filtered through a 70 μm cell strainers to collect the splenocytes.

## Antibodies Used

F4/80—biotin, clone BM8, cat # 48015 (Thermo Fisher Scientific), Anti-Biotin Microbeads, cat # 130-090-485; MS columns cat # 130-042-201 (Miltenyi Biotech, San Diego, CA), CD71—PE, C2 clone, cat # 553267 (BD Pharmigen, San Diego, CA), Ter119—PerCP Cy5.5 (clone Ter-119; cat # 45-5921-82; eBioscience, San Diego, CA), F4/80—PeCy7 (clone BM8; cat # 25-4801-82; eBioscience, San Diego, CA), ERHR3—Alexa fluor 700A; cat # MCA2393A488; Novusbio, Centennial, CO), CD169—APC (clone REA197; cat # 130-104-954; Miltenyi Biotec, San Diego, CA), CD106/VCAM1 - PacBlue, (clone 429 MVCAM.A; cat # 105722; Biolegend, San Diego, CA), Ly6G—APC Cy7 (clone 1A8; cat # 127624; Biolegend, San Diego, CA), Syto16—FITC (cat # S7578; Fischer Scientific, Waltham, MA), CD16/CD43 Mouse BD FC Block (clone 2.4G2 RUO; cat # 553141; BD Pharmingen, San Diego, CA), OneComp eBeads Compensation Beads (cat # 01-1111-41; Invitrogen/Fischer Scientific, Waltham, MA).

## Magnetic Isolation of F4/80^+^ Macrophages

Total bone marrow and spleen cells were stained with biotinylated F4/80 antibody. After 15 min of incubation at 4°C, the cells were washed and stained with anti-biotin microbeads. After 15 min of incubation at 4°C, the cells were washed and passed through the MS magnetic column (Milteneyi). F4/80^+^ cells were collected from the column using a plunger.

### Flow Cytometry

Total bone marrow cells and spleen cells were subjected to eight-color analysis (cocktail of eight antigens simultaneously); using a fluorescence activated cell-sorting (FACS) machine (LSR Fortessa 1 analyzer) and results from 150,000 events were archived. Data were obtained using Flow Jo software (Tree Star, Ashland, OR) from the viable cells gated based on forward scatter (FSC-A) and side scatter (SSC-A) and analyzed for the expression of various marker proteins using relevant antibodies as enumerated above. Positive and negative gates were set with FMO (fluorescent minus one) controls and unstained cells, respectively. Flurochrome compensation was performed using OneComp eBeads.

### Confocal Microscopy

An aliquot of TBM cells were eluted using a 21-gauge needle from untreated sham mice was stained with Siglec1-PE and Ter119-FITC. The cells were then cytospun onto microscopic slides and preserved using Vectashield H-1500 mounting medium with DAPI (Vector Laboratories, Burlingame, CA). A Zeiss LSM 510 laser-scanning microscope (Carl Zeiss MicroImaging, Jena, Germany) was used to view with C-Apochromat 403 1.20 water immersion, and X40 images were acquired using Zeiss LSM 510, version 4.2, SPI software.

### Spleen Tissue Sections

Spleens from the sham and post burn day 7 mice were isolated and fixed in 10% formalin for at least 24 h, decalcified (only femurs) and stained using hematoxylin and eosin, according to standard techniques, by the Histology Core Facility of Loyola University Medical Center. Light microscopy was performed, and digital images were captured (× 10 and × 40 magnification).

### Identification of Erythroblast Island Macrophages (EBIMØs) and Erythroblasts

A combination of markers were used to identify and quantify EBIMØ (F4/80^pos^ ER-HR3^pos^ Vcam1^pos^ Siglec1^pos^ and Ly6G^pos/neg^), total erythrons (CD71^pos^ Ter119^neg/pos^), early erythroblasts (CD71^pos^Ter119^neg^), late erythroblasts (CD71^pos^ Ter119^pos^), Orthochromatic/polychromatic erythroblasts (CD71^pos^ Ter119^pos^ Syto16^pos^), reticulocytes (CD71^pos^ Ter119^pos^ Syto16^neg^), and RBCs (CD71^neg^ Ter119^pos^) (20).

### Gating Strategy for Erythroblast Island Macrophage (EBIMØ)

To include all the macrophages that are present either in isolation or in association with erythroblasts, TBM cells and splenocytes were first gated on FCS-A and FSC-H (singlets and doublets) followed by F4/80 on x-axis and SSC-A on y-axis. Next, using histograms, we sequentially determined all the expected markers in the order of prevalence, ERHR3^pos^ >Vcam1^pos^ >Siglec1^pos^ > Ly6G^pos^. Accordingly, F4/80^pos^ cells were then gated to include ERHR3^pos^ on x-axis and F4/80^pos^ on y-axis. F4/80^pos^ ERHR3^pos^ cells were then gated on Vcam1^pos^ on y-axis and Siglec1^pos^ on x-axis. The dual positive cells predominantly constitute all EBIMØ (F4/80^pos^ ERHR3^pos^ Vcam1^pos^ Siglec1^pos^ and Ly6G^pos/neg^). We have made similar gating on F4/80^neg^ cells to confirm this gating includes most of the EBIMØs. We found there are 5,000 times more EBIMØ in F4/80^pos^ subset (see Supplemental Figure 1).

### Gating Strategy for Erythrocytes and Erythroblast Subsets

Singlets/doublets were gated on CD71 and Ter119 to identify total erythrons (CD71^pos^ Ter119^neg/pos^), which were then represented as x10^3^ cells per million TBM cells. We then re-gated the total erythrons into RBCs (CD71^neg^ Ter119^pos^), EEB (CD71^pos^ Ter119^neg^) and LEB (CD71^pos^ Ter119^pos^). There are two subsets within the LEBs; nucleated cells belong to polychromatic erythroblasts (PolyE) and orthochromatic erythroblasts (OrthoE) whereas those that lack a nucleus are reticulocytes that mature into RBCs in circulation. Therefore, we utilized the cell permeable nuclear dye Syto16 to delineate the two subsets (20). Supplemental Figure 2 compares identification of discrete erythroblasts using the above-mentioned method including Syto16 compared to the Koulnis et al. using FSC vs. CD71 (21).

### Blood Collection, Plasma Separation, and Hematrue for Blood Parameters

Peripheral blood. Heparinized blood samples from study groups were collected through cardiac puncture and examined on a veterinary hematology analyzer (HemaTrue, Heska, Love- land, CO), and RBC counts and hemoglobin levels were recorded. Plasma was separated from the blood after centrifugation at 10,000 rpm for 10 min at 4°C.

### Enzyme-Linked Immunosorbent Assay (ELISA)

G-CSF levels were analyzed as a 10x fold dilution of study plasma in Diluent A from this commercially available kit according to the manufactures' instructions (cat# EMCSF3, Thermo Scientific, Waltham, MA, USA). All measurements were preformed in duplicates.

### Statistical Analysis

Results from all experiments are expressed as mean ± sem. Number of animals used per experiment were 6–8 as given in respective figure legends. Plasma G-CSF results were determined using 4 mice per group. Analysis of variance with Tukey's *post-hoc* test using KaleidaGraph statistical program, version 4.1.0 (Synergy Software, Reading, PA, USA) were carried out for comparison between groups. Statistical significance was set at *P* < 0.05. All experiments were repeated 2–3 times.

## Results

### Medullary and Extramedullary Erythropoiesis During the Course of Burn Injury

**Figure 1:**
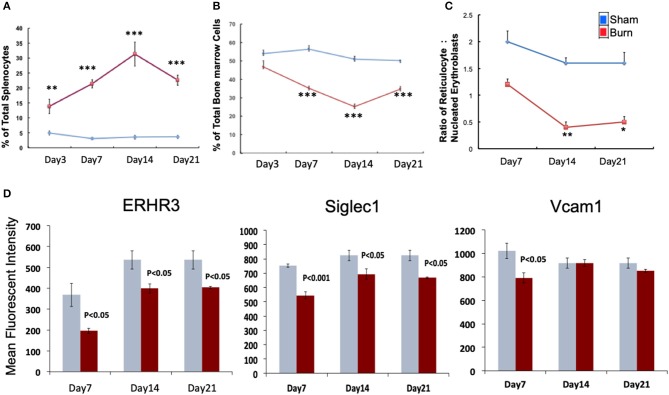
Burn injury perturbs myelopoiesis in bone marrow and spleen. **(A,B)** Line graphs represent CD71^+^Ter119^±^ cells as percentage of total spleen **(A)** and total bone marrow cells **(B)** in sham (blue) and burn injury (red) after 3, 7, 14, and 21 days. **(C)** Maturation index (MI) calculated as the ratio of reticulocytes to nucleated BM erythroblasts (represented as y-axis of the line graph) at post burn days 7, 14, and 21 (x-axis), showing a significant decrease in MI throughout the course of burn injury. **(D)** Mean fluorescent intensities of adhesion molecules ERHR3, Siglec1, and Vcam1 in F4/80^+^ MØs after 7, 14, and 21 days of burn injury are depicted as bar graphs (sham = blue/gray, burn = red). Reduced expression of the three adhesion molecules is seen at post burn day 7. Error bars indicate standard error of mean of >3 independent experiments. ^*^P < 0.05, **P < 0.001; ***P < 0.0001 vs. burn by one-way ANOVA.

We monitored the medullar and extra medullar erythropoietic responses from days 3 through 21 post burn. While extra medullar erythropoiesis was active through the course of burn injury starting from day 3, medullar erythropoiesis was significantly attenuated from day 7 as measured by the percentage of CD71^+^Ter119^±^ cells in spleen and TBM. The ratio of reticulocytes to nucleated BM erythroblasts (maturation index) was significantly decreased from day 7 through day 21 as an indication of late maturation defects following burn injury. Further, during the same study period, F4/80^+^ MØ in the BM showed deficiency in MØ associated adhesion molecule expression (Figures 1A–D). Therefore, we chose day 7 post burn to study the phenotype of EBIMØs and the stage specific association of erythroblasts in a comprehensive manner during medullar arrest and extra medullar excess in BM and spleen, respectively, to observe the status of EBI integrity.

## Bone Marrow

### Bone Marrow Erythroblast Island Macrophage Phenotype From a Macrophage Perspective

**Figure 2:**
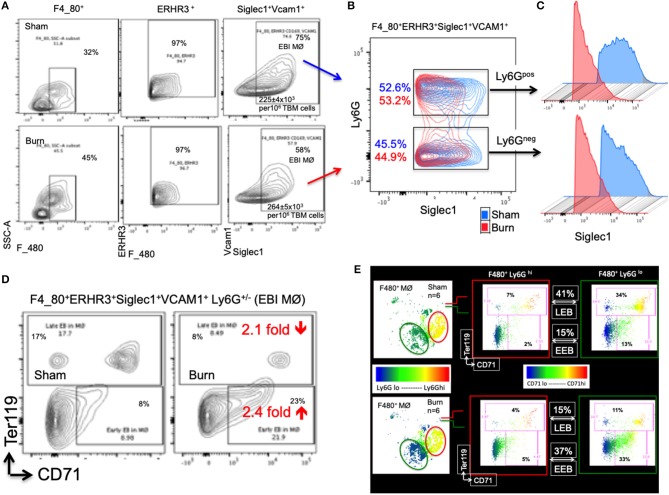
Characterization of erythroblast island forming macrophages (EBIMØs) in sham and burn mice. **(A)** EBIMØs in total bone marrow (TBM) cells isolated from sham and post burn day 7 (PBD7) mice were characterized by the presence of cell surface markers-F4/80, ERHR3, Siglec1, Vcam1, and Ly6G. F4/80^+^ERHR3^+^Siglec1^+^Vcam1^+^Ly6G^±^ macrophages constituted the EBI forming population as analyzed by flow cytometry. **(B)** Overlay of EBIMØs contour plots (Sham = blue, PBD7 = red) gated on Siglec1 and Ly6G shows equal distribution between Ly6G^pos^ and Ly6G^neg^ cells. **(C)** Histogram of Siglec1 expression as measured by MFI (X-axis) in EBIMØs of burn (red) compared to Sham (blue) from both the Ly6G ^pos^ and Ly6G ^neg^ subsets **(D)** The EBIMØs were analyzed for the presence of erythroblast (EB) markers. CD71^+^Ter119^−^ cells represent early EBs (EEBs) and CD71^±^Ter119^+^ cells represent late EBs (LEBs). **(E)** Data from 6 animals were analyzed using t-SNE plots to find the association of Ly6G with EBs. F4/80^+^ MØs were gated on Ly6G marker (color scale blue to red indicates Ly6G low to high subsets) was checked for CD71 (X-axis) and Ter119 (Y-axis) expressions in sham (top panel) and PBD7 (bottom panel) mice. Ly6G low subset was more associated with EB markers when compared to Ly6G high subset in both the treatment groups. Among the F4/80^+^Ly6G^lo^ MØs in sham, there were 3 times more LEB and 2.5 times less EEB association compared to the burn group. Data represent mean values containing 6 mice/group.

The percentage of EBIMØ was determined for each sample as explained in the methods section and back-calculated to represent absolute numbers in a million TBM cells. The total number of EBIMØ per million TBM cells was marginally higher in burn group compared to sham (S = 225 ± 4.0 × 10^3^, B = 264 ± 5.4 × 10^3^; *p* < 0.002; Figure 2A). As this EBIMØ gating includes both Ly6G^pos^ and Ly6G^neg^ cells, we further gated these MØ on Ly6G and Siglec1 expressions. While there was an equal distribution of Ly6G^pos^ and Ly6G^neg^ MØs in this fraction (Figure 2B), we noticed a significant reduction in Siglec1 expression in EBIMØ from burn compared to sham (S = 2,977 ± 58, B = 1,913 ± 71; *p* < 0.0001; Table 1). Moreover, we noticed a 40% reduction in Siglec1 expression irrespective of Ly6G^pos^ or Ly6G^neg^ EBIMØ from burn mice (Histograms in Figure 2C). We then documented the distribution of erythroblasts associated with these EBIMØ. As shown by contour plots in Figure 2D, there was a striking contrast in the type of erythroblasts nurtured by bone marrow EBIMØs from sham vs. burn mice. More EEBs from burn mice and more LEBs from sham mice were associated with EBIMØ. The mean fluorescent intensities of Ly6G, ERHR3, and VCAM1 expressions were similar between sham and burn EBIMØ isolated from the femurs (Table 1). These results still do not answer the importance of Siglec1 or Ly6G on EBIMØ.

**Table 1:** F4/80^+^ERHR3^+^Siglec1^+^Vcam1^+^Ly6G^±^ EBIMØ phenotype (Mean fluorescence intensity).

	**ERHR3**	**Vcam1**	**Siglec1**	**Ly6G**
**Bonemarrow**				
Sham	475 ± 16	772 ± 15	2,977 ± 58	1,704 ± 45
Burn	528 ± 20	791 ± 24	1,913 ± 71^***^	1,491 ± 100
**Spleen**				
Sham	465 ± 28	660 ± 32	965 ± 37	357 ± 47
Burn	572 ± 23^*^	713 ± 45	927 ± 33	624 ± 68^**^

* p < 0.02,

** p < <0.01,

*** *p < 0.0001 vs. Sham EBIMØ*.

### Is Ly6G Expression Critical for Bone Marrow EBIMØ Association With Early and Late Erythroblasts?

To inquire the importance of Ly6G and its association with EEB and LEB in F4/80^pos^ MØ, we utilized a machine-learning algorithm called T-distributed stochastic neighbor embedding (t-SNE) plots as shown in Figure 2E. Overall, comparing Ly6G^lo^ vs. Ly6G^hi^ MØ, Ly6G^lo^ expressing MØ were predominantly associated with EEBs and LEBs irrespective of sham or burn conditions. Nonetheless, among the F4/80^pos^Ly6G^lo^ MØs, we noticed 3 times more LEB association in sham and 2.5 times more EEB association in burn groups.

### Bone Marrow Total Erythrons and Erythroblast Subsets Are Altered After Burn

**Figure 3:**
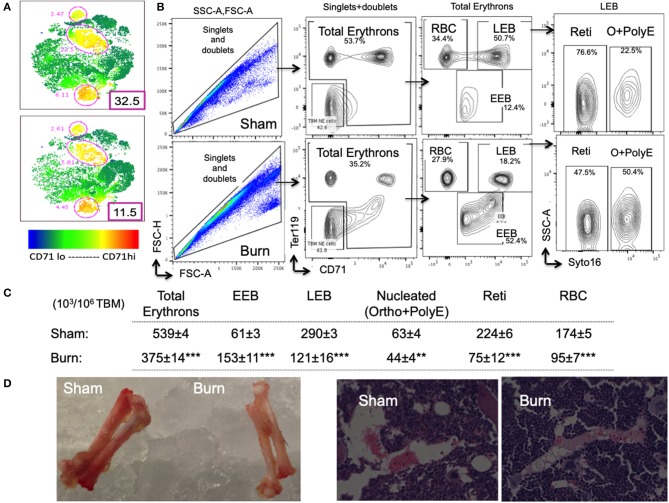
Decline in late erythroblasts and reticulocytes with an increase in early erythroblasts after burn injury. **(A)** t-SNE plots showing a decrease in CD71 expression in total bone marrow (TBM) erythroid cells in post burn day 7 (PBD7) vs. sham mice. **(B)** During flow cytometry data analysis using FlowJo software, both singlets and doublets from total bone marrow (TBM) cells were selected to quantitate total erythrons. This cell population was then gated on early erythroid marker CD71 on X-axis and Ter119 (mature erythroid cell marker) on Y-axis. Dual negative cells were gated as TBM non-erythroid (NE) cells and the rest of the cells as total erythrons, which were significantly decreased in post burn day 7 (PBD7) mice. Total erythrons were sub-gated as Ter119^−^CD71^+^ early erythroblasts (EEBs) and Ter119^+^CD71^+^ late erythroblasts (LEBs). A strikingly high percentage of EEBs was observed in the PBD7 samples accompanied by a decrease in LEBs. LEBs were further classified as the nucleated ortho (O) and polychromatic erythroblasts (PolyEs) and the enucleated reticulocytes (Reti) based on the Syto16 nuclear staining. A stagnation of nucleated erythroblasts was seen in the PBD7 TBM. **(C)** Absolute numbers of erythron subsets per million of TBM cells in sham vs. burn injury are given. **(D)** Isolated femurs from sham and burn mice and H&E staining of paraffin embedded sections are shown. Pale color and myeloid enriched bone marrow is evident in the burn-injured mice compared to sham respectively. Data are representative of 6 independent experiments. Data represent mean ± SEM, containing 6 mice/group and repeated 4 times. **P < 0.002; ***P < 0.0001 sham vs. burn; comparison by one-way ANOVA.

For overall comparison between two groups and to visualize flow data, we utilized t-sne plots denoting CD71^+^ TBM cells. Bone marrow CD71^lo−hi^ cells are a representation of erythroid staining obtained from six animals per group shown in t-sne plots (Figures 3A,B). We noticed a 2.8-fold decrease in the percentage of CD71^hi^ bone marrow cells from burn group.

As expected, total erythrons present in a million TBM cells were decreased by 44% in burn group compared to sham (S = 539 ± 3.5 × 10^3^, B = 375 ± 14 × 10^3^, *p* < 0.0001). Following burn injury; total RBCs, reticulocytes, and nucleated orthochromatic and polychromatic erythroblasts were significantly decreased. Amongst the erythroblast developmental stages, EEB and LEB subsets were associated with EBIMØ at a ratio of 2:1 and 5:1, respectively in both groups (S vs. B is not significant). However, there were more EEBs per million TBM cells in burn group (S = 61.2 ± 2.5 × 10^3^, B = 153 ± 10.7 × 10^3^; *p* < 0.0001). On the other hand, we noticed a 2.4-fold decrease in the number of LEBs in burn group (S = 290 ± 7 × 10^3^, B = 121 ± 16 × 10^3^; *p* < 0.0001). Figure 3C enumerates the distribution of erythroblast subsets calculated as × 10^3^/10^6^ TBM cells. Isolated femurs and H&E staining of paraffin embedded sections reveal overall bleaching of femurs on day 7 after burn injury (Figure 3D). Bone marrow paraffin sections were subjected to IHC using Ter119-FITC a pan erythroid marker to further confirm significant reductions in RBC after burn (Supplemental Figure 3).

### Stage Specific Preference for Association With EBIMØ by Bone Marrow Erythroblasts: Validated From an Erythroblast Perspective

**Figure 4:**
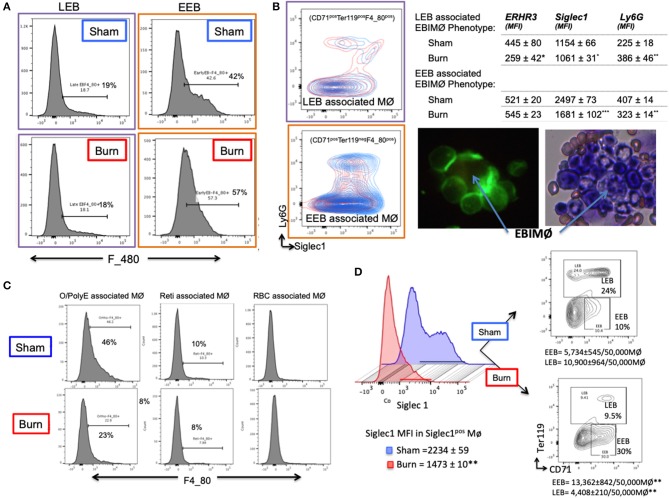
Erythroblast associated macrophages are also altered after burn injury. **(A)** Histograms show the expression of macrophage (MØ) marker F4/80 on early and late erythroblasts (EBs) indicating the association pattern with EBIMØs. Late EBs (LEBs) expressed higher F/480 expression compared to early erythroblasts (EEBs). **(B)** Representative contour plots of EEB and LEB associated macrophages for Siglec1 and Ly6G markers in sham and PBD7 groups are shown (sham = blue, burn = red). Adjacent panel shows the mean fluorescent intensity of ERHR3, Siglec1, and Ly6G in the EBIMØ associated with LEB and EEB. Significantly lower Siglec1 and Ly6G MFI was observed in EEBs of burn mice compared to sham. In burn LEBs, the decrease in Siglec1 MFI is not as comparable as seen in EEBs, probably indicating weaker association with MØs as the EBs mature from early to late stage. Confocal image shows EBI with a central MØ (magnetic isolation of F4/80^+^ MØ) surrounded by erythroblasts (green). Image depicting a central MØ surrounded by early erythroblasts in the proximity and more mature reticulocytes in the vicinity as identified by Maygrunwald Giemsa staining of eluted BM. **(C)** LEBs were divided into nucleated EBs (O/PolyE) and enucleated reticulocytes (Reti) and RBCs to analyze the associated MØs as represented in the histograms. Only the nucleated EBs showed F4/80 expression whereas <10% enucleated EBs expressed F4/80 marker. Data represent 6 independent biological replicates and error bars indicate standard error of the mean. **(D)** Histograms represent Siglec-1 intensity of magnetically sorted F4/80^pos^ MØ from BM of Sham (blue) and Burn (red) mice. Association of early and late erythroblasts with the sorted macrophages were determined by plotting on CD71 (X-axis) and Ter119 (Y-axis) to show that stagnation of EEBs in burn mice is associated with 1.4-fold down regulation of Siglec1.*P < 0.05, **P < 0.02; ***P < 0.0001 sham vs. burn by one-way ANOVA.

Based on the results that EEBs are more ubiquitous in burn than sham mice (Figures 3B,C), then the EEBs from burn mice should show similar association with EBIMØ phenotype expressing less Siglec1 compared to sham EBIMØ (Figure 2C). We then determined whether or not erythroblast dependency on EBIMØ varies with the developmental stages of erythroblast subsets. To answer these, we probed from the erythroblast perspective with the notion that if the erythroblasts were associated with MØ, then remnants of MØ marker F4/80 should still be expressed by the erythroblast of interest. We confirmed this notion by imaging stream-using AMNIS (Supplemental Figure 4). Therefore, EEB and LEB (OrthoE/PolyE), were re-gated for F4/80 expression, which indicates those erythroblast subtypes that were associated with bone marrow MØ (Figure 4A).

Macrophages associated with either EEB or LEB from sham and burn mice were analyzed for EBIMØ phenotype based on Siglec1 and Ly6G expressions (Figure 4B). We noticed significantly lower Siglec1 and Ly6G in EEB associated MØ from burn mice (Siglec-1: S = 2,497 ± 73, B = 1,681 ± 102; *p* < 0.0001. ERHR3: S = 521± 20, B = 545 ± 23; ns; Ly6G: S = 407 ± 14, B = 323 ± 14; *p* < 0.02). In LEB associated MØ, Siglec-1 and ERHR3 expressions were decreased, while Ly6G expression was increased (Siglec-1: S = 1154 ± 66, B = 1061 ± 31; *p* < 0.05. ERHR3: S = 445 ± 80, B = 259 ± 42; *p* < 0.05; Ly6G: S = 225 ± 18, B = 386 ± 46; *p* < 0.05). The other MØ associated marker VCAM1 was not altered with burn injury.

As shown in the histogram (Figure 4C), only EEB and Ortho/PolyE (nucleated LEB subset) were predominantly associated with F4/80^pos^ macrophages whereas, the enucleated reticulocytes and mature RBCs were not associated with F4/80^pos^ macrophages. This observation was consistent in both groups showing no variations with burn injury.

In magnetically sorted F4/80^pos^ macrophages (Figure 4D), we saw similar associations between low Siglec1 expression and more EEBs in Burn mice compared to Shams (Burn: MFI = 1,473 ± 10 and EEB = 13,362 ± 842/50,000 F4/80^pos^ MØ; Sham: MFI = 2,234 ± 59 and EEB = 5,734 ± 545/50,000 F4/80^pos^ MØ; *p* < 0.001). On the contrary, association of LEBs was significantly higher in F4/80^pos^ MØ isolated from the BM of Sham compared to Burn mice (10,900 ± 964/50,000 vs. 4,408 ± 210/50,000 F4/80^pos^ MØ; *p* < 0.001).

## Spleen

### Splenic Erythroblast Island Macrophage Phenotype After Burn Injury From a Macrophage Perspective

**Figure 5:**
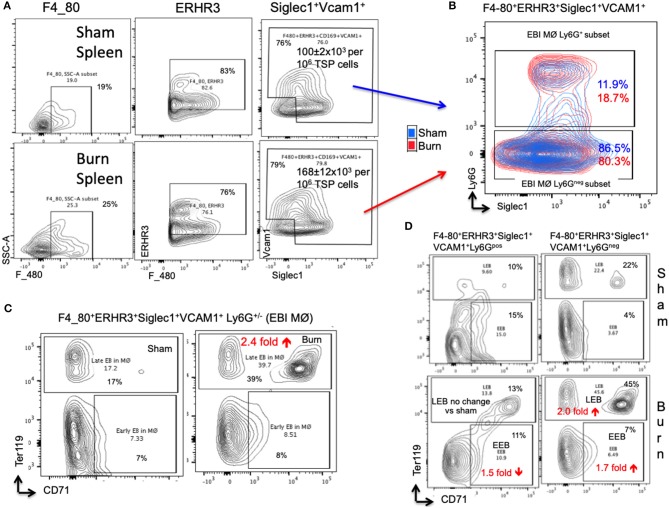
Erythroblast island (EBI) profile in spleen is a mirror image of bone marrow seen after burn injury. **(A)** Erythroblast island (EBI) profile was characterized in spleen by plotting the total spleen (TSP) cells on F4/80, ERHR3, Siglec1, and Vcam1 markers in both sham and PBD7 treatment groups. A marked increase in absolute number of EBIMØs (ERHR3^+^Siglec1^+^Vcam1^+^F4/80^+^ Ly6G^±^) was observed in burns compared to sham. **(B)** An exemplary superimposed contour plot image of splenic EBIMØ from sham (blue) and burn (red) gated on Ly6G (Y-axis) and Siglec1 (X-axis). **(C)** The EBIMØs were examined for erythroblast (EB) markers CD71 and Ter119. A 75% increase in late EBs (LEBs) was observed in the spleen following burn injury. **(D)** Contour plots demonstrate the analysis of F4/80^+^ ERHR3^+^ Siglec1^+^ Vcam1^+^ EBIMØs based on Ly6G expression and their correlation with erythroblasts (EBs). LEBs were mostly associated with Ly6G low subset in both sham and burn groups, however, more LEBs were in association with Ly6G^low^ EBIMØs in the burn group. Data represent mean ± SEM, containing 6 mice/group and repeated 4 times.

In the spleen, the number of EBIMØs per million splenocytes were significantly higher in burn group compared to sham as shown in Figure 5A (S = 100 ± 2 × 10^3^, B = 168 ± 12 × 10^3^; *p* < 0.002). As this gating includes both Ly6G^pos^ and Ly6G^neg^ EBI MØ, we further gated these MØ on Ly6G and Siglec1. Overall, there were more Ly6G^neg^ than Ly6G^pos^ EBIMØ in the spleen compared to BM (Figure 5B). Nonetheless, we found a significant increase in ERHR3 and Ly6G expression in EBIMØ from burn spleen (MFI of ERHR3: S = 465 ± 28, B = 572 ± 23; *p* < 0.02; Ly6G: S = 357 ± 48, B = 624 ± 67; *p* < 0.01) compared to sham. More importantly, EBIMØ from spleen exhibited an increase in Siglec-1 and Vcam1 expressing F480^+^ macrophages after burn injury (Table 1). Interestingly, examining the erythroblasts that are associated with EBIMØ in spleen following burn injury revealed a 75% increase in LEBs compared to sham (Figure 5C).

### Is Ly6G Expression Critical for Splenic Erythroblast Island Macrophage EBIMØ Association With Early and Late Erythroblasts?

Based on the above observation, which was in contrast to bone marrow results, we examined the requirement of Ly6G expression in EBIMØ in spleen especially with respect to EEBs and LEBs. Given that the expression of Ly6G is higher in EBIMØ from burn mice compared to sham, the proportion of Ly6G^pos^ EBIMØ were only marginally greater in burn spleens (Figure 5B). But, there was no change in LEBs associated with these MØ between the two conditions. In fact, we saw a 1.5-fold decrease in EEBs associated with Ly6G^pos^ EBIMØ from burn vs. sham spleens. As seen with bone marrow, only Ly6G^neg^ EBIMØ were associated with LEBs both in sham and burn spleens (Figure 5D).

### Total Erythrons and Erythroblast Subsets in the Spleen Are Increased After Burn

**Figure 6:**
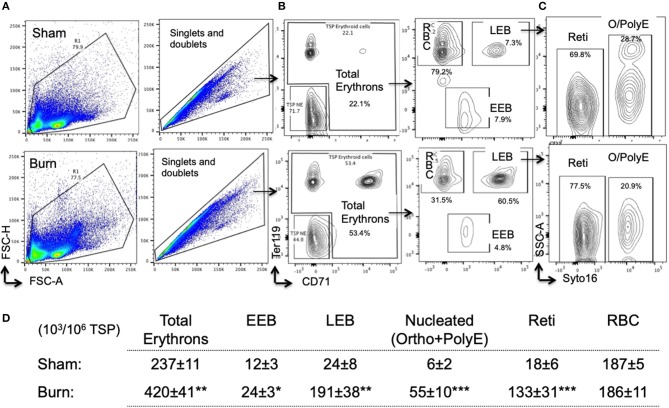
Erythropoiesis is enhanced in spleen after burn injury synchronizing with EBIMØ association with late erythroblasts. **(A)** Total spleen cells (TSP) was gated for combined singlets and doublets based on FSC-A vs. FSC-H. **(B)** TSP were then gated on early erythroid marker CD71 (X-axis) and mature erythroid cell marker Ter119 on (Y-axis). Dual negative cells were gated as TSP non-erythroid (NE) cells and the rest of the cells as total erythrons, which were significantly increased in post burn day 7 (PBD7) mice. Total erythrons were sub-gated as Ter119^−^CD71^+^ early erythroblasts (EEBs) and Ter119^+^CD71^+^ late erythroblasts (LEBs). **(C)** LEBs were further classified as the nucleated ortho (O) and polychromatic erythroblasts (PolyEs) and the enucleated reticulocytes (Reti) based on the Syto16 nuclear staining. **(D)** The numbers of erythron subsets per million of TSPs in sham vs. burn injury. Total erythron as well as all erythron subsets-EEBs, LEBs, nucleated EBs (ortho and polychromatic EBs) and enucleated EBs (reticulocytes and RBCs) were significantly increased in PBD7 vs. sham. Data represent mean ± SEM, containing 6 mice/group and repeated 4 times. *P < 0.002, **P < 0.006; ***P < 0.0003 vs. sham by one-way ANOVA.

Overall, total erythrons in spleen are increased by 75% following burn. While there was no change in mature RBCs between sham and burn, all other erythroblast developmental stages were significantly increased indicating robust extra medullary erythropoiesis after burn (Figures 6A–D).

**Figure 7:**
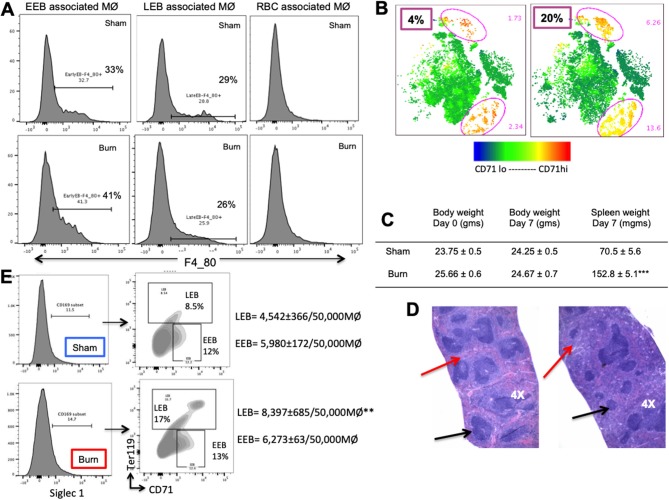
Phenotypic changes accompany hematopoietic alterations in spleen after burn injury. **(A)** Splenic erythroblasts (EBs) were analyzed for MØ marker F4/80 to see the association with erythroblast island macrophages (EBIMØs) in a stage specific manner, as represented in the flow cytometry histograms. EBIMØs are required by only early erythroblasts (EEBs) and late EBs (LEBs) and not by mature RBCs. **(B)** t-SNE plots showing the distribution of CD71^+^ cells in sham and burn mice. Marked increase in CD71^+^ cells were observed in the spleen after burn injury (represented by yellow cell population). **(C)** Table showing the change in body weight and spleen weights in sham vs. post burn day 7 mice. Weight of spleens increased significantly in the burn condition, showing splenomegaly to support extramedullary hematopoiesis. **(D)** H&E staining of paraffin embedded sections of the isolated spleens. Black arrows point to the white pulp and red arrows to the red pulp. Splenomegaly and increase in the red marrow is observed on day 7 after burn injury compared to sham spleens. **(E)** Magnetically sorted splenic F4/80^+^ MØ from Burn mice are represented in histogram with Siglec1 expression on the X-axis. F4/80^+^ MØ were then plotted on CD71 (x-axis) and Ter119 (y-axis). Sorted PBD7 splenic macrophages have similar siglec1 expression and associated EEBs, but higher LEBs compared to Sham. Data and pictures are representative of mean ± SEM, containing 6 mice/group. **P < 0.01, ***P < 0.0001 vs. sham by one-way ANOVA.

Moreover, EBIMØ dependency of splenic erythroblasts is stage specific, very similar to bone marrow erythroblasts. EBIMØs are required by only EEBs and LEBs and not by mature RBCs (Figure 7A). Erythroid cell (CD71) distribution pattern in spleen is provided as t-SNE plots in Figure 7B. Splenomegaly is evident in mice subjected to burn injury supporting extra medullar erythropoiesis, with no change in total body weight between the cohorts on post burn day 7 (Figure 7C). H&E staining of paraffin embedded sections of spleens on day 7 after burn injury showed evidence of extra-medullary hematopoiesis compared to Shams as noted by the diffused expansion of the red pulp characterized by a morphological predominance of nucleated erythroid precursors (Figure 7D). In magnetically sorted F4/80^pos^ macrophages from the spleens (Figure 7E), we saw no change in Siglec1 expression by MØ in Burn mice compared to Shams. Contrary to BM, association of LEBs was significantly higher in F4/80^pos^ MØ isolated from the spleen of Burn compared to Sham mice (8,397 ± 685/50,000 vs. 4,542 ± 366/50,000 F4/80^pos^ MØ; *p* < 0.001), while that of EEBs were similar in both groups.

## Peripheral Blood Profile and Plasma G-CSF Levels

Plasma G-CSF was below detectable limits in the Sham group; reached highest levels on post burn day 3 (PBD 3) (833 ± 214 pg/ml) and dropped significantly by PBD 7 (217 ± 39 pg/ml). Granulocyte numbers followed the same pattern as G-CSF with PBD 3 exhibiting higher percentage (39 ± 2.29%; *p* < 0.001) compared to sham (28.28 ± 4.86%) and PBD 7 values (29.7 ± 0.75%). On the other hand, RBCs, Hb, and HCT were not altered on PBD 3, but were significantly decreased on PBD 7 (Table 2).

**Table 2:** Blood parameters.

	**Sham**	**PBD 3**	**PBD 7**
Plasma G-CSF (pg/ml)	ND	833 ± 214^**^	217 ± 39^∧^
Granulocytes (%)	28.28 ± 4.86	39.38 ± 2.29^*^	29.70 ± 0.75^∧^
RBC (10^6^/μl)	10.41 ± 0.20	9.90 ± 0.13	8.98 ± 0.16^***^^∧^
Hb (g/dl)	15.45 ± 0.26	14.83 ± 0.23	13.15 ± 0.24^***^^∧^
HCT (%)	46.97 ± 0.82	45.22 ± 0.50	40.63 ± 0.79^***^^∧^

*ND, Below detection limits of the ELISA kit*.

*** p < 0.0002,

** p < 0.002,

* p < 0.001 vs. Sham;

∧ *p < 0.01 vs. PBD 3. One way ANOVA*.

**Figure 8:**
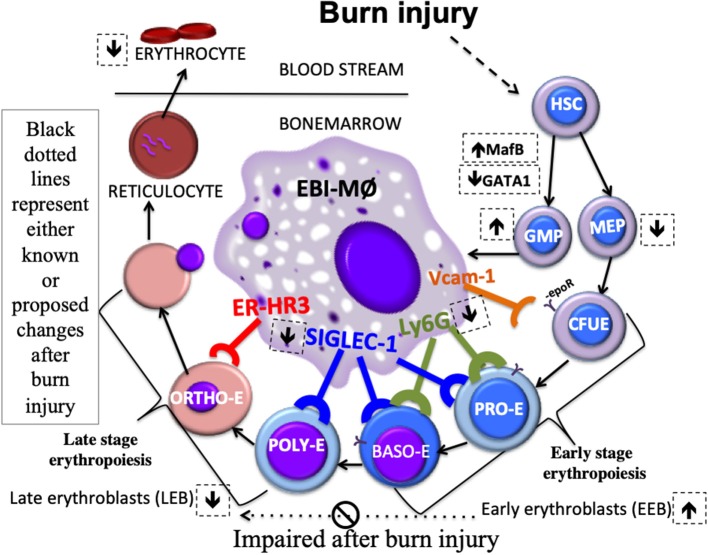
Schematic Abstract. Macrophages stemming from a burn-induced microenvironment exhibit altered phenotype of EBI macrophages. EEBs are tightly associated with EBIMØ than LEBs. Siglec1 and Ly6G are predominantly required by these EBIMØ for effective differentiation of EEBs into LEBs. It is conceivable that down-regulated Siglec1 are associated with the decrease in LEBs in burn conditions partly contributing to anemia of critical illness.

As depicted in the schematic (Figure 8), we state that myeloid cells stemming from a burn-induced microenvironment will impede red blood cell development, conceivably by the altered phenotype of EBI forming macrophages. Moreover, EEBs are tightly associated with EBIMØ than LEBs and that Siglec1 and Ly6G are predominantly required by these EBIMØ for effective differentiation of EEBs into LEBs.

## Discussion

Here, we have elucidated that burn injury perturbs bone marrow EBIMØ phenotype. Further, Siglec1 down-regulation emerges as a predominant deficit in EBIMØ after burn from both the erythroblast as well as the MØ perspective. In contrast to bone marrow EBIMØ, Ly6G, and ERHR3 are stronger in splenic EBIMØ while Siglec1 expression is not reduced in response to burn injury, sparing extra medullary erythropoiesis presenting the possibility of the interference of myeloid abnormality disrupting early to late erythroblast maturation.

The higher percentage of EEBs (2.5-fold) associated with low Siglec1 expressing EBIMØ after burn injury could probably mean an impaired differentiation to LEBs because, LEBs are also found to be reduced in the bone marrow of mice subjected to burns with a parallel increase in EEBs (2.4-fold) resulting in overall 45% reduction in total erythrons in line with our earlier report (20). Lack of MØ availability can be ruled out because there were no differences between sham and burn in the ratio of EEB and LEB to EBIMØ at 2:1 and 5:1, respectively. This could imply a specific role for Siglec1 during the process of differentiation from proerythroblasts to PolyE, OrthoE, and reticulocyte stages. One eminent function is to provide iron to the attached EEBs as EBIMØ have been shown to make iron available during erythroblast maturation (22), but do not specify the role of Siglec1 in this process. Alternatively, one can speculate, Siglec1 is important for chromatin condensation or for the formation of contractile actin rings involved in nuclear pyknosis, all of which are needed during erythroblast maturation (23). This speculation is further strengthened by our results from spleen where Siglec1 expression is maintained, and extramedullary response is robust, with more LEBs (2.4-fold) that are associated with EBIMØ, resulting in a 70% increase in overall splenic erythrons, with significant increases in EEBs (30%) and LEBs (75%) after burn.

Splenic responses to thermal injury have previously been reported when granulocytes, granulocyte-macrophages, and macrophage progenitors increase soon after thermal injury (14% TBSA), with maximal responses seen after a week (24). In the same study they showed an increase in the number of marginal zone (CD115^+^) and not red pulp (F4/80^+^) macrophages after thermal burn, which matches with our current observation of insignificant increase in percentage of splenic F4/80^+^ MØ on day 7 after burn (15% TBSA). Nonetheless, alterations in MØ phenotypes are quite possible in other models of burn injury as shown by an increase in CD11b^+^F4/80^+^ peritoneal macrophages (25).

Splenic erythropoiesis is increased at the expense of medullar erythropoiesis when erythroid island macrophages were depleted simultaneously with G-CSF mobilization (14). Our results are supported by the above study because, we observed similar BM erythroid arrest in our model and additionally, burn patients have increased plasma levels of catecholamines and G-CSF (26) echoing a plausible G-CSF mediated response. Moreover, in the same study, CD169 specific depletion of MØ in G-CSF treated mice resulted in significant loss of reticulocytes with corresponding increase in the number of pro erythroblasts (14). Similarly, our model of burn injury resulted in high numbers of stagnant EEBs after burn, with significant reduction in LEBs and reticulocytes, further strengthening our notion that late maturation arrest is evident in burn-induced anemia.

In order to address the influence of G-CSF in burn mediated erythroid arrest, we measured progressive changes in plasma G-CSF levels and also peripheral blood parameters at post burn day 3 (PBD) and PBD 7. Because we used 10x dilutions of plasma, Sham levels of G-CSF were below detection limits. However, peak levels at PBD 3 is in line with our groups previous reports (27). Interestingly, these levels were reduced by PBD 7, which explains why peripheral blood granulocytes follow the same pattern while red blood cell parameters like RBC numbers, Hb, and Hct begin to decline on PBD 7 in line with our previous observations (4, 20). Of note G-CSF is only transiently elevated in burn patients compared to pro-inflammatory cytokines (28). Perhaps, burn mediated pro-inflammatory cytokines such as TNF-alpha, IL-1β, IL-6, and IFN-gamma could influence impaired erythropoiesis as in Hep3B cells (29) and engineered stromal cells in BM cultures (30). On the other hand, Means et al. have shown that TNF inhibits bone marrow CFU-E indirectly through stromal elements, which is abrogated by antibodies to human IFN-beta, but not by antibodies to IFN-gamma or IL-1 (31). Aside from these arguable preclinical data that inflammatory signaling pathways, such as TNF-alpha, IL-1β, IL-6, and IFN-gamma, can negatively influence Epo-dependent/independent erythropoiesis, their clinical translation has been challenging in humans and needs in depth investigations.

Glucocorticoid receptor (GR) signaling has been shown to induce monocyte differentiation that shares the phenotypical characterization of EBIMØs (32). However, in the setting of burn injury, GR expression and binding capacity were increased in circulating leukocytes compared to healthy subjects (33) eliminating the probability of GR signaling as Siglec1 is down regulated in EBIMØ after a burn injury.

While studies characterizing the EBIMØs is ongoing (6, 7, 15, 34, 35), Jacobsen et al. have recently reported a combination of markers namely, CD11b^+^F4/80^+^ Vcam1^+^ER-HR3^+^ Siglec1^+^Ly6G^+^ as signatures that represent EBIMØ in BM and spleen. Our report does not completely agree with their signature because, in our hands, association of EEBs and LEBs with EBIMØs are independent of Ly6G expression following burn injury both in BM and spleen. This discrepancy in LY6G expression is probably representation of non-erythroid cells by the inclusion of CD11b^+^F4/80^+^ MØ in Jacobson's characterization of EBIMØ. As suggested by Seu et al. using multispectral imaging, while CD11b, albeit abundantly expressed by cells within islands, is not expressed on the EBIMØ (17). We have also noticed the possibility of multi lobular myeloid cell interference adjacent to intact erythroblast islands (EBIs) amongst nucleated erythroblasts (data not presented). Besides, the predominant requirement of Siglec1 in EBIMØ reported in our current study is further supported by Seu et al.'s observation that more than 90% of F4/80^+^ EBIMØs were also positive for CD169 (Siglec1), unlike CD11b, which was not co-expressed with F4/80^+^ MØ. Nonetheless, we have taken both LY6G^pos^ and LY6G^neg^ cells into account for calculating the total number of EBIMØs eliminating any disparity in characterizing overall BM response.

Reports from the same study show no significant changes in F4/80 and CD169 expressing splenocytes after the induction of stress erythropoiesis. Our results also indicate that Siglec1 (CD169) expression is not altered in spleen after burn injury, and the major difference in EBIMØ phenotype between BM and spleen is a BM specific 40% decrease in Siglec1 expression. Moreover, results from magnetically sorted F4/80 ^pos^ MØ from the BM reinforce that when Siglec1 expression is down regulated (by 1.5-fold), about 2.3-fold more EEBs and 2.5-fold less LEBs are associated with them. Therefore, higher LEB and lower EEB counts associated with sham macrophages indicate a high turnover rate of EEB to LEB differentiation. On the contrary, lower LEB and higher EEB counts associated with burn macrophages indicate a dampened turnover rate of LEB to EEB differentiation. Whereas, no difference in either Siglec1 expression or EEB association from splenic F4/80 ^pos^ MØ with two times more LEB association in Burn compared to Sham further strengthens our notion that EBIMØ from spleen and BM are not the same in our burn injury model. Therefore, it is plausible that the hampered progress from early to late stage erythroblasts is coupled with Siglec1^lo^ EBIMØ impeding BM erythropoiesis. This observation is consistent with a previous study done in the model of phenylhydrazine (PHZ)-induced stress erythropoiesis in which the authors found an expansion of F/480 cells expressing Vcam1 and CD169 markers (36). From another point of view, one can argue that EEBs express receptors for erythropoietin (Epo-R) and therefore Epo-R mediated signaling mechanisms can be implicated in maturation arrest (37). Moreover, erythropoietin rescues G-CSF induced CFU-E arrest in naïve and splenectomized mice (38). We acknowledge while this is a possibility, considering burn patients have normal to high erythropoietin levels (1), Epo-dependent erythropoiesis stages are intact in burn injured subjects (39), and that higher doses of Epo promotes migration of BFU-E from BM to spleen preserving robust proliferation (40), Epo-R defect is highly unlikely as splenic erythropoiesis is augmented in our current study. Nonetheless, our focus is to decipher myeloid derived EBIMØ phenotypic alterations resulting from burn injury independent of Epo-R signaling.

In hematopoietic stem and progenitor cell (HSPC) mobilization studies, G-CSF can independently increase BM sympathetic tone by increasing nor-epinephrine availability (41). While Siglec1 is presumed to be involved in EBI forming MØs (6), retention of hematopoietic stem cells (HSCs) and erythroid progenitors in the BM is dependent on macrophages expressing Siglec1 (42). Since sympathetic nervous system (SNS) responses via beta2 and beta3 adrenergic signals are implicated in HSC mobilization (43–45), we can speculate that enhanced sympathetic tone following burn (26) may explain the reduction in Siglec1 expression in BM EBIMØ. We have previously shown that burn injury significantly increased the LSK positive cells in the BM (4) and non-specific beta-blockade after burn was effective in partially mitigating this response (46). Nonetheless, in burn patients, late-stage erythropoiesis remain impaired irrespective of propranolol treatment implying the possibility of beta-2 independent terminal maturation defects (9). Additionally, our finding of beta3-antagonist -mediated rescue of late erythropoiesis in burn injured mice (20) supports our hypothetical notion of beta3-adrenergic regulation of Siglec1^+^ EBIMØ. However, studies using specific pharmacologic antagonists to beta2- and beta3-adrenergic receptors are required to delineate the dual mechanisms down regulating Siglec1 in EBIMØ following burn, which is a major limitation of the current manuscript. Further, identification of those intercellular signals that enhance positive interactions between EEBs and Siglec1^++^ EBIMØs to augment their maturation to LEBs will remain a key issue for future studies.

Given the intricacies in obtaining the erythroblast island without compromising its integrity, this is our preliminary study observing the down regulation of specific macrophage-associated adhesion molecule that is perturbed by burn injury plausibly impeding erythropoiesis. Our results emphasize transient increase in G-CSF cannot drive this persistent anemia in burn patients, Therefore, based on the persistent increase in catecholamines in burn patients and our preliminary data with beta 2 and 3 adrenergic blockade (47) we speculate that sympathetic stimulation of beta3 adrenergic receptors could play a predominant role in burn induced EBIMØ phenotype. While current study establishes the premise, targeted knock down of Siglec1 in bone marrow macrophages or studies in beta-3 adrenergic knock out mice are required to further expand our observations, which is beyond the scope of the current manuscript.

Here we have shown that only nucleated erythroblasts (early and late) are tightly associated with EBIMØ (F4/80^+^ERHR3^+^VCAM1^+^Siglec1^+^LY6G^±^), where Siglec1 and Ly6G are predominantly required for EBI integrity in the BM. Burn injury significantly diminishes the expression of Siglec1 in BM EBIMØs suggesting a role for the myeloid derived MØ in the maturation of pro erythroblasts into poly/orthochromatic erythroblasts and reticulocytes. Having established this methodology to ascertain stage specific dependence of erythroblast maturation on EBIMØ phenotype, detailed functional and mechanistic studies utilizing Siglec1^−/−^ and or anti-GCSF and beta3 antagonist treatment in mice subjected to burn injury will be the focus of our future studies.

## Data Availability Statement

The raw data supporting the conclusions of this manuscript will be made available by the authors, without undue reservation, to any qualified researcher.

## Ethics Statement

This study was carried out in accordance with the recommendations of AALAC guidelines by the IACUC committee. The Institutional Animal Care and Use Committee at Loyola University Medical Center approved all experimental protocols.

## Author Contributions

KM designed, analyzed, interpreted, and wrote the paper. SH conducted experiments and analyzed and wrote parts of paper. MJ carried out some experiments with revision and helped with the response to reviewers' comments. AK interpreted histopathology results and provided consultation. AB provided clinical consultation and reviewed the manuscript in preparation.

### Conflict of Interest

The authors declare that the research was conducted in the absence of any commercial or financial relationships that could be construed as a potential conflict of interest.

## Supplementary Material

Supplemental Figure 1 An exemplary F4/80^pos^ (red) and F4/80^neg^ (blue) fraction of TBM cells were gated to show ERHR3 CD169 (Siglec1), and Vcam1 in histograms. MFI of each adhesion molecule is shown in the legend below respective histogram overlay. Also EBIMØ counts between F480^pos^ and F480^neg^ fractions are shown in the contour plots with F480 on X-axis and CD169 (Siglec1) on Y axis.

Supplemental Figure 2 Gating strategy to compare erythroblasts populations by two different flow cytometry-based approaches. **(A)** Singlets from total bone marrow cells were gated on Ter119 (X-axis) and CD71 (Y-axis) and selected for Ter119^neg^ ProEs and Ter119^+^ cells. Ter119^+^ cells were then gated on FSC-A (X-axis) and CD71 (Y-axis). Based on CD71 intensity and size (FSC-A), erythroblast subsets were divided into EryA (CD71^+^, FSC-A high); EryB (CD71^+^, FSC-A low); and EryC (CD71^neg^, FSC-A low). **(B)** Singlets from total bone marrow cells were gated on CD71 (X-axis) and Ter119 (Y-axis) and selected for Ter119^neg^ ProEs and Ter119^+^ cells were gated as CD71^neg^ Ter119^+^ RBCs and CD71^+^ Ter119^+^ late erythroblasts (LEBs). LEBs were further sorted on the basis of Syto16, CD71^+^ Ter119^+^ Syto16^+^ cells were categorized as Poly and Orthochromatic erythroblasts, and CD71^+^ Ter119^+^ Syto16^neg^ cells as reticulocytes. Comparing **(A,B)**, it is clear that 1. ProE and EEB are one and the same 2. EryC and RBC are one and the same and 3. EryA and EryB have some overlapping nucleated and enucleated erythroblasts whereas in B there is clear demarcation. This validates our current method is superior in identifying discrete populations.

Supplemental Figure 3 Immunofluorescence microscopy on paraffin embedded sections of femurs obtained from sham and burn mice using Ter119-FITC, a pan erythroid marker to further confirm significant reductions in RBC after burn.

Supplemental Figure 4 AMNIS Imagestream showing the association of erythroblasts with Siglec-1 positive macrophages.

## Abbreviations

BM: Bone marrow
EBI: Erythroblast Island
EBIMØ: Erythroblast Island macrophages
EEB: Early erythroblasts
LEB: Late erythroblasts
MØ: Macrophage
OrthoE: Orthochromatophilic erythroblasts
PolyE: Polychromatophilic erythroblasts
Reti: Reticulocytes
TBM: Total bone marrow cells
TBSA: Total body surface area
TSP: Total Spleen Cells.
