# Supplementary figures and images for: A Shift in Myeloid Cell Phenotype via Down Regulation of Siglec-1 in Island Macrophages of Bone Marrow Is Associated With Decreased Late Erythroblasts Seen in Anemia of Critical Illness
Source: Front Med (Lausanne). 2019 Nov 20;6:260. doi: 10.3389/fmed.2019.00260 (PMC6880610; doi:10.3389/fmed.2019.00260)

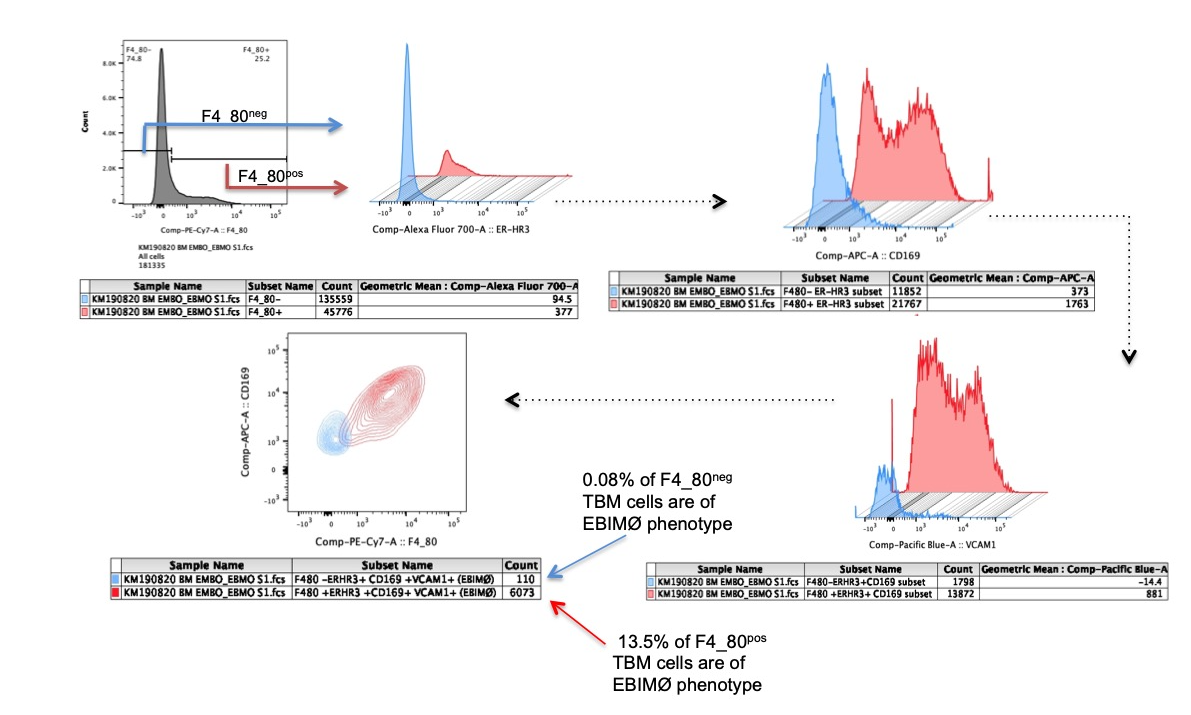

Supplement: Supplemental Figure 1 — An exemplary F4/80pos (red) and F4/80neg (blue) fraction of TBM cells were gated to show ERHR3 CD169 (Siglec1), and Vcam1 in histograms. MFI of each adhesion molecule is shown in the legend below respective histogram overlay. Also EBIMØ counts between F480pos and F480neg fractions are shown in the contour plots with F480 on X-axis and CD169 (Siglec1) on Y axis. [file Image_1.tiff]

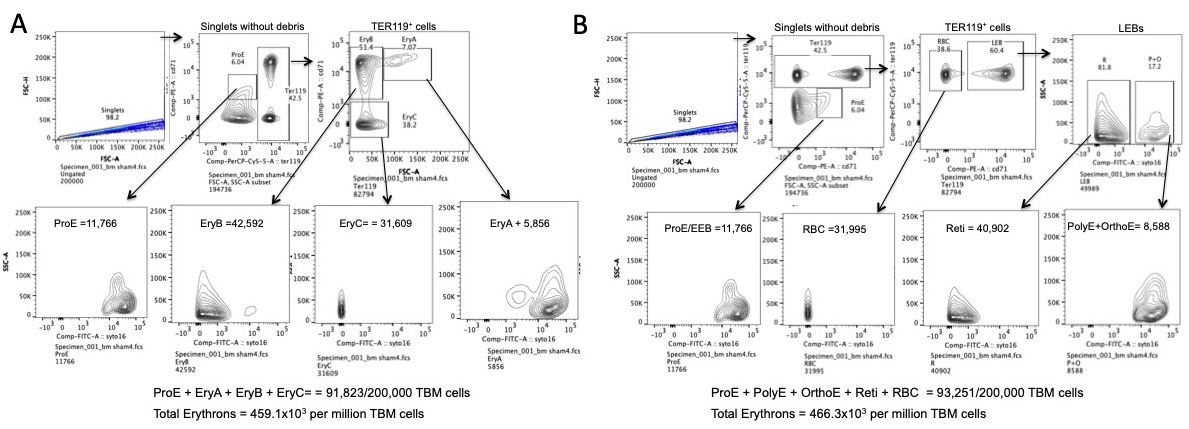

Supplement: Supplemental Figure 2 — Gating strategy to compare erythroblasts populations by two different flow cytometry-based approaches. (A) Singlets from total bone marrow cells were gated on Ter119 (X-axis) and CD71 (Y-axis) and selected for Ter119neg ProEs and Ter119+ cells. Ter119+ cells were then gated on FSC-A (X-axis) and CD71 (Y-axis). Based on CD71 intensity and size (FSC-A), erythroblast subsets were divided into EryA (CD71+, FSC-A high); EryB (CD71+, FSC-A low); and EryC (CD71neg, FSC-A low). (B) Singlets from total bone marrow cells were gated on CD71 (X-axis) and Ter119 (Y-axis) and selected for Ter119neg ProEs and Ter119+ cells were gated as CD71neg Ter119+ RBCs and CD71+ Ter119+ late erythroblasts (LEBs). LEBs were further sorted on the basis of Syto16, CD71+ Ter119+ Syto16+ cells were categorized as Poly and Orthochromatic erythroblasts, and CD71+ Ter119+ Syto16neg cells as reticulocytes. Comparing (A,B), it is clear that 1. ProE and EEB are one and the same 2. EryC and RBC are one and the same and 3. EryA and EryB have some overlapping nucleated and enucleated erythroblasts whereas in B there is clear demarcation. This validates our current method is superior in identifying discrete populations. [file Image_2.tiff]

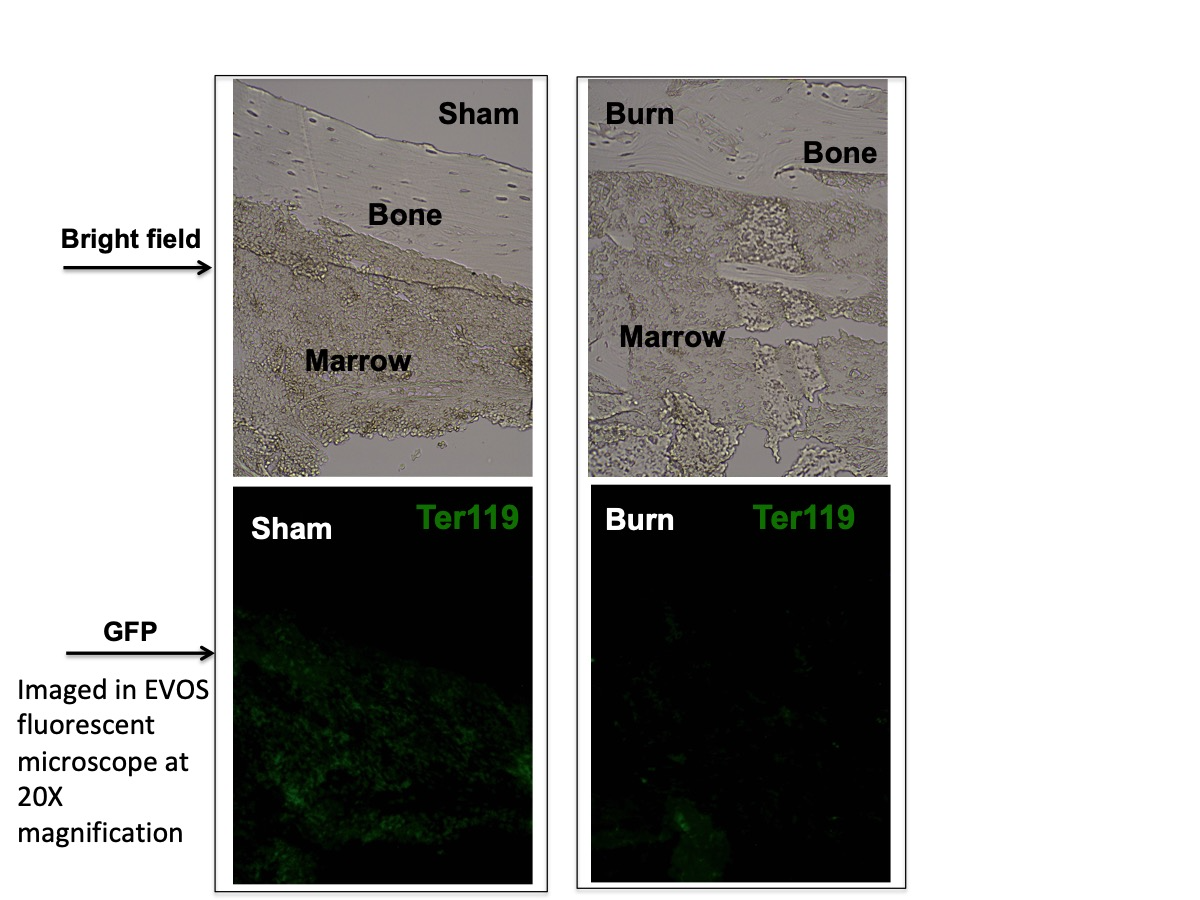

Supplement: Supplemental Figure 3 — Immunofluorescence microscopy on paraffin embedded sections of femurs obtained from sham and burn mice using Ter119-FITC, a pan erythroid marker to further confirm significant reductions in RBC after burn. [file Image_3.tiff]

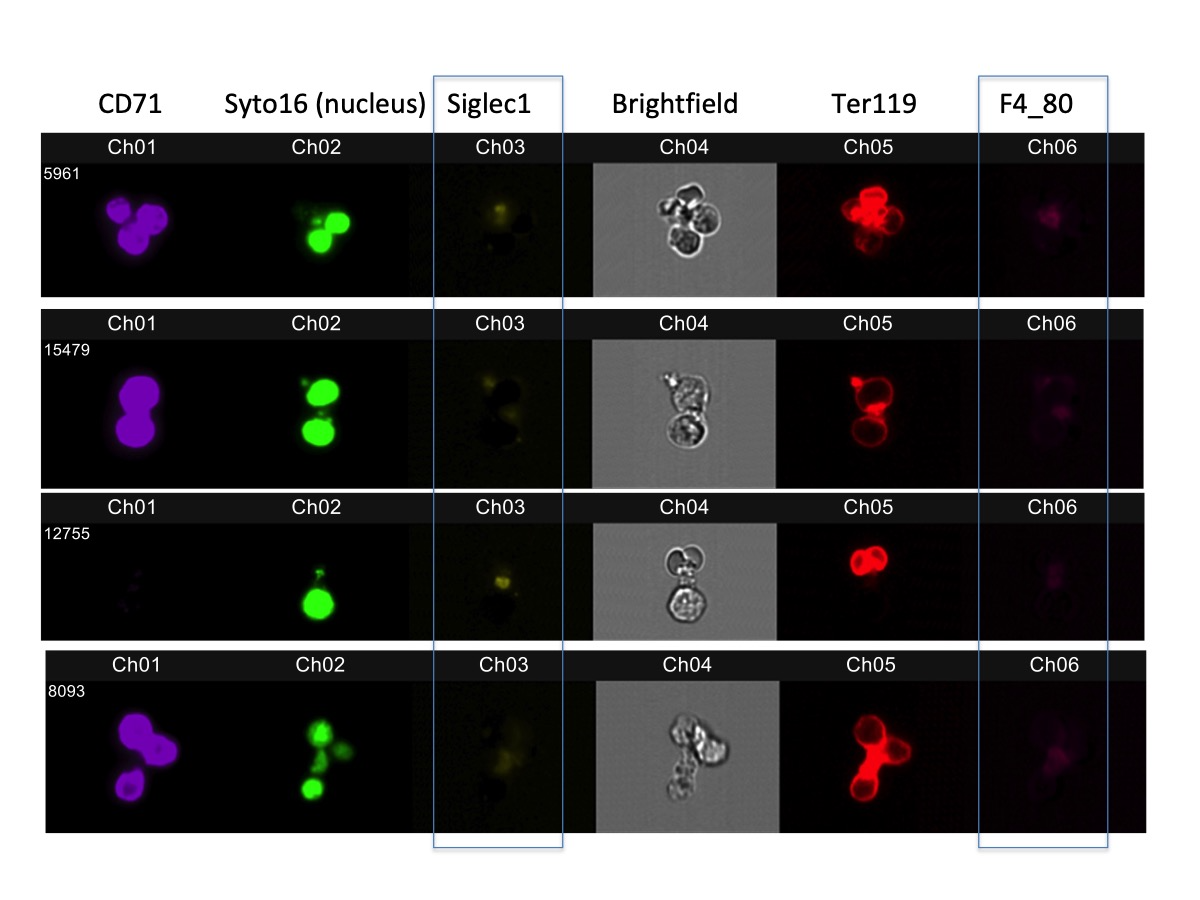

Supplement: Supplemental Figure 4 — AMNIS Imagestream showing the association of erythroblasts with Siglec-1 positive macrophages. [file Image_4.tiff]
